# Volumetric three-dimensional intravascular ultrasound visualization using shape-based nonlinear interpolation

**DOI:** 10.1186/1475-925X-12-39

**Published:** 2013-05-07

**Authors:** Yonghoon Rim, David D McPherson, Hyunggun Kim

**Affiliations:** 1 Department of Internal Medicine, Division of Cardiovascular Medicine, The University of Texas Health Science Center at Houston, 6431 Fannin St, MSB 1.246, Houston, TX 77030, USA

**Keywords:** Intravascular ultrasound, Shape-based interpolation, Three-dimensional reconstruction

## Abstract

**Background:**

Intravascular ultrasound (IVUS) is a standard imaging modality for identification of plaque formation in the coronary and peripheral arteries. Volumetric three-dimensional (3D) IVUS visualization provides a powerful tool to overcome the limited comprehensive information of 2D IVUS in terms of complex spatial distribution of arterial morphology and acoustic backscatter information. Conventional 3D IVUS techniques provide sub-optimal visualization of arterial morphology or lack acoustic information concerning arterial structure due in part to low quality of image data and the use of pixel-based IVUS image reconstruction algorithms. In the present study, we describe a novel volumetric 3D IVUS reconstruction algorithm to utilize IVUS signal data and a shape-based nonlinear interpolation.

**Methods:**

We developed an algorithm to convert a series of IVUS signal data into a fully volumetric 3D visualization. Intermediary slices between original 2D IVUS slices were generated utilizing the natural cubic spline interpolation to consider the nonlinearity of both vascular structure geometry and acoustic backscatter in the arterial wall. We evaluated differences in image quality between the conventional pixel-based interpolation and the shape-based nonlinear interpolation methods using both virtual vascular phantom data and *in vivo* IVUS data of a porcine femoral artery. Volumetric 3D IVUS images of the arterial segment reconstructed using the two interpolation methods were compared.

**Results:**

*In vitro* validation and *in vivo* comparative studies with the conventional pixel-based interpolation method demonstrated more robustness of the shape-based nonlinear interpolation algorithm in determining intermediary 2D IVUS slices. Our shape-based nonlinear interpolation demonstrated improved volumetric 3D visualization of the *in vivo* arterial structure and more realistic acoustic backscatter distribution compared to the conventional pixel-based interpolation method.

**Conclusions:**

This novel 3D IVUS visualization strategy has the potential to improve ultrasound imaging of vascular structure information, particularly atheroma determination. Improved volumetric 3D visualization with accurate acoustic backscatter information can help with ultrasound molecular imaging of atheroma component distribution.

## Background

Intravascular ultrasound (IVUS) is a standard imaging modality at the time of vascular interventions to identify pathologic alterations of atherosclerotic plaque by evaluating acoustic backscatter information using the cross-sectional arterial image data [1-3]. In most IVUS imaging protocols, a series of cross-sectional two-dimensional (2D) IVUS images is recorded along the longitudinal direction while the IVUS catheter is being withdrawn. This provides challenges in concurrent evaluation of multiple regions of interest (ROIs) over the arterial structure [3]. Since atherosclerosis is a diffuse disease and atherosclerotic plaques can vary markedly along the arterial structure, clinicians need to clearly evaluate the type and morphology of atherosclerotic plaques [4]. Three-dimensional (3D) IVUS visualization can provide these data.

Current 3D IVUS imaging methods are performed using two techniques. The first and most popular method is to simply generate a pixel-based cut-view image of the artery showing acoustic backscatter information within the arterial wall along the blood flow direction [5]. This method, however, only creates another 2D image only inside the arterial wall along the longitudinal direction, and thus cannot provide comprehensive 3D information pertaining to spatial atheroma distribution over the entire arterial structure. The other popular 3D IVUS imaging technique is to focus on creating anatomically realistic luminal contour borders using algorithms for smooth 3D surface rendering [6]. However, detailed acoustic backscatter information across the arterial wall is discarded with this methodology when surface rendering is performed for 3D visualization.

Several studies have reported 3D reconstruction algorithms for 3D IVUS imaging to better provide anatomical information and overcome the limited 3D visualization capability of currently available commercial systems [3,6-8]. Previous studies to overcome this problem include utilization of conventional intermediary slice generation method [3], and shape-based interpolation algorithms [9]. These studies still provide sub-optimal visualization of arterial morphology and lack acoustic information concerning arterial structure due in part to low quality of image data and the use of pixel-based IVUS image reconstruction algorithms.

Since the distance between neighboring pixels in each 2D IVUS image is much smaller than the distance between the 2D IVUS images along the longitudinal direction leading to non-isotropic voxel dimensionality, 3D IVUS algorithms often yield deterioration of image quality following 3D reconstruction [10]. It is expected that increased discretization between 2D IVUS images combined with a proper interpolation method can provide improved volumetric 3D IVUS visualization.

We have recently demonstrated a volumetric 3D IVUS visualization methodology for early and inflammatory arterial atheroma characterization [11]. These volumetric 3D IVUS images provide 3D arterial structure visualization as well as acoustic backscatter distribution information for plaque and atheroma determination. In this volumetric 3D IVUS reconstruction algorithm, 2D IVUS image (i.e., pixelated) data with a conventional pixel-based IVUS image reconstruction method were only utilized. In the present study, we describe an improved 3D IVUS algorithm using IVUS signal data and a shape-based nonlinear interpolation method to generate intermediary slices between the original 2D IVUS slices and create volumetric 3D IVUS images in an *in vivo* model.

## Methods

**Figure 1:**
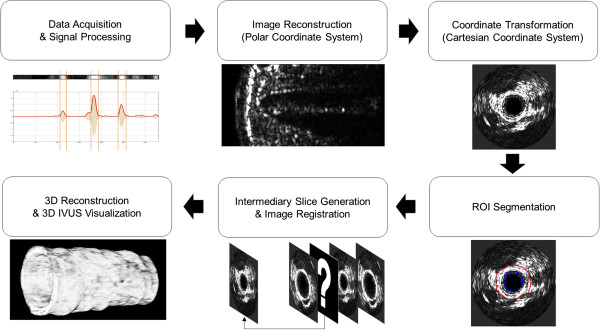
Protocol of the volumetric 3D IVUS visualization strategy using the shape-based nonlinear interpolation method.

We developed an algorithm to convert a series of IVUS signal data into a fully volumetric 3D visualization (Figure 1). The entire protocol including 2D image reconstruction from IVUS signal data, border tracing, segmentation, sequential alignment of 2D IVUS slices, and intermediary slice generation was conducted in a single image processing platform. Volumetric visualization of 3D IVUS images was performed using ImageJ, open-source Java-based image processing software provided by the National Institutes of Health.

### IVUS signal data acquisition *in vivo*

An atherosclerotic Yucatan miniswine atheroma model (20 kg, Sinclair Research Center Inc., Columbia, MO) was used. The animal protocol was approved by the Institutional Animal Care and Use Committee of The University of Texas Health Science Center at Houston. Following anesthesia, the right femoral artery was exposed with groin incisions, and an arteriotomy was performed. A 5 F sheath was inserted into the femoral artery. A high frequency (20 MHz, 3.5 F) IVUS imaging catheter connected to a Volcano s5i IVUS Imaging System (Volcano Co, Rancho Cordova, CA) was utilized. The IVUS catheter was inserted through the arterial segment past the region of interest, and withdrawn using an automatic pullback device at a constant speed of 0.5 mm/s, while 2D IVUS images and signal data of the arterial segment were continuously recorded. A total of 256 scan lines with 1,024 sampling data per scan line were recorded (dynamic range of 40–60 dB) for each 2D IVUS slice. Since there was no curvature in the arterial segment evaluated, it was assumed that the direction of pullback of the IVUS catheter was parallel to the longitudinal direction of the artery.

The enveloped amplitude (i.e., acoustic backscatter) was utilized to reconstruct images in the polar coordinate, in which the x- and y- axes refer to the radial and circumferential directions, respectively. The reconstructed images in the polar coordinate were transformed to the Cartesian coordinate system for standard vascular imaging. A graphical user interface (GUI)-based image processing system was developed for interactive tracing and segmenting procedures under MATLAB (Mathworks Inc., Natick, MA) platform. The inner and outer arterial borders in each IVUS image were manually segmented. A series of signal data in the segmented ROIs was placed in tomographic sequence to create intermediary slices.

We collected a total of 25 original 2D IVUS slices of the arterial segment with a distance of 0.5 mm between slices to generate 10 intermediary slices between two adjacent slices resulting in a total of 265 cross-sectional images along the longitudinal direction. In order to interpolate IVUS signal data of the arterial segments in 3D space, we utilized the natural cubic spline interpolation considering the nonlinearity of both vascular structure geometry and acoustic backscatter information within the arterial wall.

### Intermediary IVUS slice generation using the shape-based nonlinear interpolation

**Figure 2:**
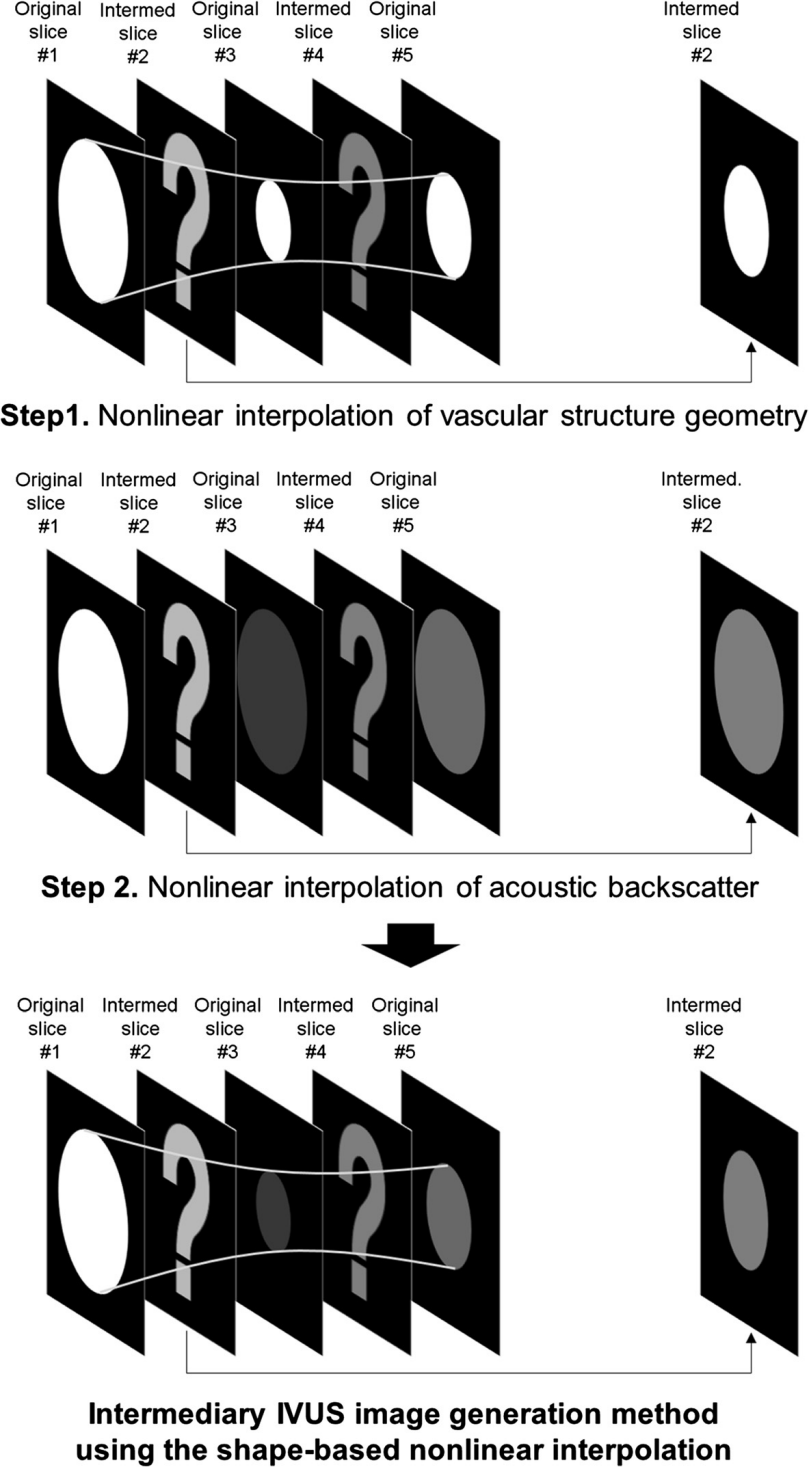
Schematic diagram of the intermediary IVUS image generation algorithm utilizing both arterial geometry and acoustic backscatter information in the shape-based nonlinear interpolation method.

Figure 2 demonstrates a schematic diagram of the intermediary IVUS slice generation algorithm using the shape-based nonlinear interpolation method. Intermediary slices between original 2D IVUS slices are generated utilizing both arterial geometry and acoustic backscatter information using vascular geometry information in the three neighboring slices (Step 1). We applied the natural cubic spline interpolation method to the segmented ROIs of 2D IVUS data sequentially placed along the longitudinal direction. The contours delineating arterial wall structure in the three neighboring 2D IVUS slices were utilized to calculate inner and outer arterial borders in intermediary slices along the 256 scan lines. Next, nonlinear acoustic backscatter interpolation was performed (Step 2). The segmented ROI of the arterial wall in each IVUS slice contains acoustic backscatter profile. This information was utilized to interpolate acoustic backscatter distribution within the ROIs in the consequent intermediary slices using the natural cubic interpolation.

**Figure 3:**
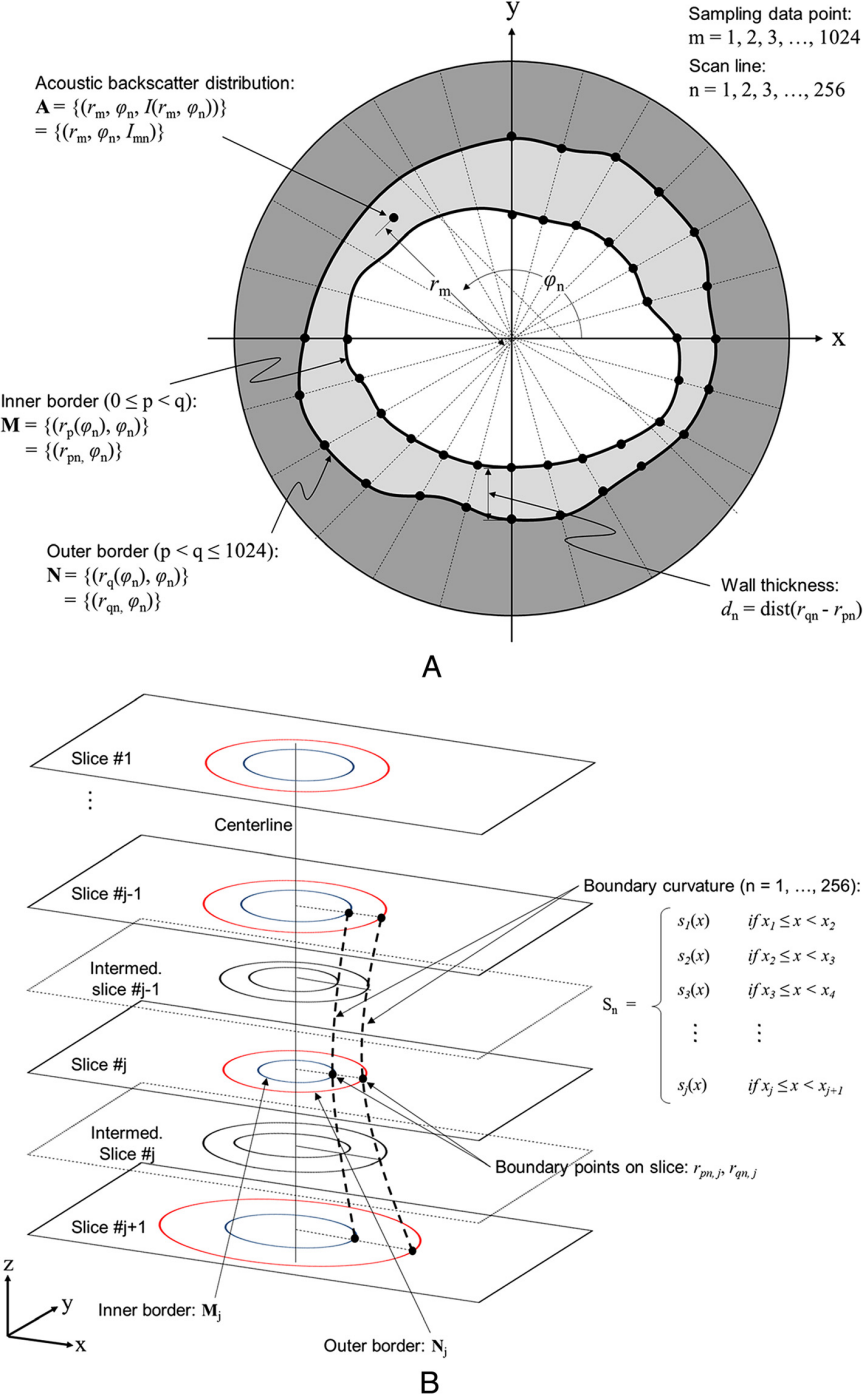
**Variables, functions and conditions utilized in the shape-based nonlinear interpolation method. (A)** Basic parameters in the polar coordinate system. (**B**) Algorithm of the shape-based nonlinear interpolation to create the inner and outer boundaries and acoustic backscatter information in the intermediary slices.

Figure 3A demonstrates variables, functions and conditions utilized in the shape-based nonlinear interpolation method. Acoustic backscatter information within the segmented ROI was defined as **A** = {(*r*_m_, *φ*_n_, *I*(*r*_m_, *φ*_n_))} = {(*r*_m_, *φ*_n_, *I*_mn_)} along the scan lines bounded by the inner arterial structure border contour **M** = {(*r*_p_(*φ*_n_), *φ*_n_)} = {(*r*_pn_, *φ*_n_)} and the outer border contour **N** = {(*r*_q_(*φ*_n_), *φ*_n_)} = {(*r*_qn_, *φ*_n_)} in each 2D IVUS image in the polar coordinate system. In this configuration, the radial distance *r* is defined as a function of *φ* which is the angular position of the scan line. There are a total of 256 scan lines (n) with 1,024 sampling data (m) per scan line.

Detailed information of the shape-based nonlinear interpolation algorithm is described in Figure 3B. First, the original 2D IVUS slices were registered in the global coordinate and the centroid of the arterial structure on each slice was aligned to the centerline. The inner boundary point on the *n*^*th*^ scan line in the *j*^*th*^ slice was defined *r*_*pn,j*_ = (*x*_*j*_, *r*_*j*_)^T^. In order to calculate the coefficients of the polynorms that constitute boundary splines of the *n*^*th*^ scan line along the longitudinal direction using *j + 1* number of slices, the boundary points data were defined as (*x*_*1,*_*r*_*1*_),…, (*x*_*j+1,*_*r*_*j+1*_).

For any *i* ∈ {*1,…, n*}, we set *z*_*i*_ *= s″*(*x*_*i*_) and Δ *x*_*i*_ *= x*_*i+1*_*- x*_*i*_. For all *j* ∈ {*1,…, n - 2*} and *x* ∈ [*x*_*j*_*, x*_*j+1*_], the second derivative of a cubic spine function *s*_*j*_*″*(*x*) is the line connecting (*x*_*j,*_*z*_*j*_) and (*x*_*j+1,*_*z*_*j+1*_). The equation is given by

(1) $${s^{\prime \prime }}_{j}\left(x\right)=\frac{x-x_{j}}{\Delta x_{j}}z_{j+1}-\frac{x-x_{j+1}}{\Delta x_{j}}z_{j}$$

By integrating (1), we have

(2) $${s^{\prime }}_{j}\left(x\right)=\frac{1}{2}{\left(x-x_{j}\right)}^{2}\frac{z_{j+1}}{\Delta x_{j}}-\frac{1}{2}{\left(x-x_{j+1}\right)}^{2}\frac{z_{j}}{\Delta x_{j}}+B_{j}$$

For (*A*_*j*_*, B*_*j*_) ∈ **R**^2^, we have

(3) $$s_{j}\left(x\right)=\frac{{\left(x_{j+1}-x\right)}^{3}}{6\Delta x_{j}}z_{j}+\frac{{\left(x-x_{j}\right)}^{3}}{6\Delta x_{j}}z_{j+1}+A_{j}+B_{j}\left(x-x_{j}\right)$$

Given with *s*(*x*_*j*_) = *r*_*j*_ and *s*(*x*_*j+1*_) = *r*_*j+1*_, the coefficients of the polynorms can be calculated as

(4) $$A_{j}=r_{j}-\frac{z_{j}{\left(\Delta x_{j}\right)}^{2}}{6}\;\mathrm{and}\;B_{j}=\frac{\Delta r_{j}}{\Delta x_{j}}-\frac{\Delta z_{j}\Delta x_{j}}{6}$$

The continuity of *s″* in *x*_*1*_ and *x*_*n*_ implies that

(5) $$z_{1}=z_{n}=0$$

For all *j* ∈ {*2,…, n – 1*}, the continuity of *s′* in *x*_*j*_ implies that

(6) $$\frac{\Delta x_{j-1}}{6}z_{j-1}+\left(\frac{\Delta x_{j}}{3}+\frac{\Delta x_{j-1}}{3}\right)z_{j}+\frac{\Delta x_{j}}{6}z_{j+1}=\frac{\Delta r_{j}}{\Delta x_{j}}-\frac{\Delta r_{j-1}}{\Delta x_{j-1}}$$

A system of linear equations (6) represented by a tridiagonal matrix can be employed to calculate the spline interpolation functions in equation (3) to define the inner boundary curvature between the slices. Likewise, the outer boundary curvature was determined.

Next, we created the same number of acoustic backscatter data points between the two borders with an arterial wall thickness *d*_n_ = dist(*r*_qn_ - *r*_pn_) on each scan line. Following the inner and outer boundaries defined on the intermediary slices as above, the acoustic backscatter within the ROI in the intermediary slices were determined across 100 data points for each scan line using the same natural cubic interpolation algorithm.

### Validation studies of the shape-based nonlinear interpolation

**Figure 4:**
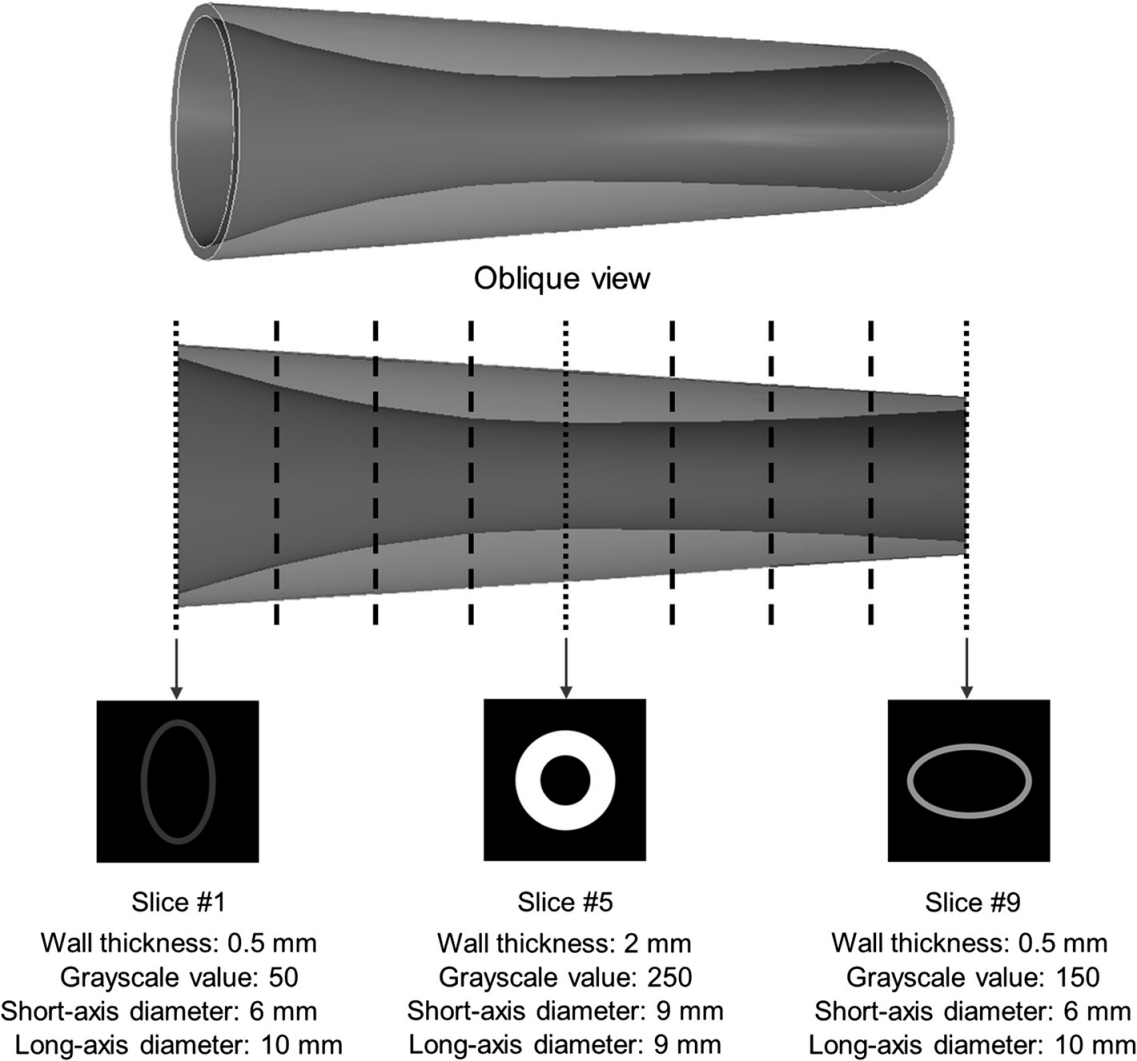
**A virtual vascular phantom containing cross-sectional images with varying shapes, wall thicknesses and grayscale values.** Three slices (#1, #5, #9) were utilized as original input slices.

Our shape-based nonlinear interpolation is developed to create intermediary slices incorporating both geometric interpolation and grayscale interpolation. In order to validate our shape-based nonlinear interpolation algorithm, we utilized a virtual vascular phantom containing cross-sectional images with varying shapes, wall thicknesses and grayscale values (Figure 4). Three cross-sectional images (#1, #5, #9) were utilized as input data to calculate three intermediary slices between each pair. Detailed dimensional information of the three original input slice images are described in Figure 4. We compared geometry and grayscale information of the intermediary slices created by the shape-based nonlinear interpolation with the original cross-sectional images at the corresponding location of the phantom. Next, we evaluated differences in image quality between the conventional pixel-based interpolation and the shape-based nonlinear interpolation methods using both the phantom data and the femoral artery IVUS data.

### Volumetric 3D IVUS visualization of the femoral artery

We created 10 intermediary slices for every pair of 25 original IVUS slices using the conventional pixel-based interpolation and our shape-based nonlinear interpolation methods. This permitted evaluation of a total of 265 IVUS images along the longitudinal direction of the femoral artery. Image stocks containing 265 IVUS slice images created from each interpolation method were utilized for volumetric 3D IVUS visualization. The stacks were imported to the volume viewer in ImageJ. In order to better demonstrate the difference between the two interpolation methods, we displayed both inner and outer surfaces of the arterial segment.

In order to demonstrate the practical applicability of our shape-based nonlinear interpolation method to 3D IVUS reconstruction along curved pullback trajectories (e.g., coronary arteries), we created a curved trajectory (s-shaped with one third height with respect to the arterial segment length) and incorporated the same IVUS data into the trajectory.

## Results

### Validation studies of the shape-based nonlinear interpolation

**Figure 5:**
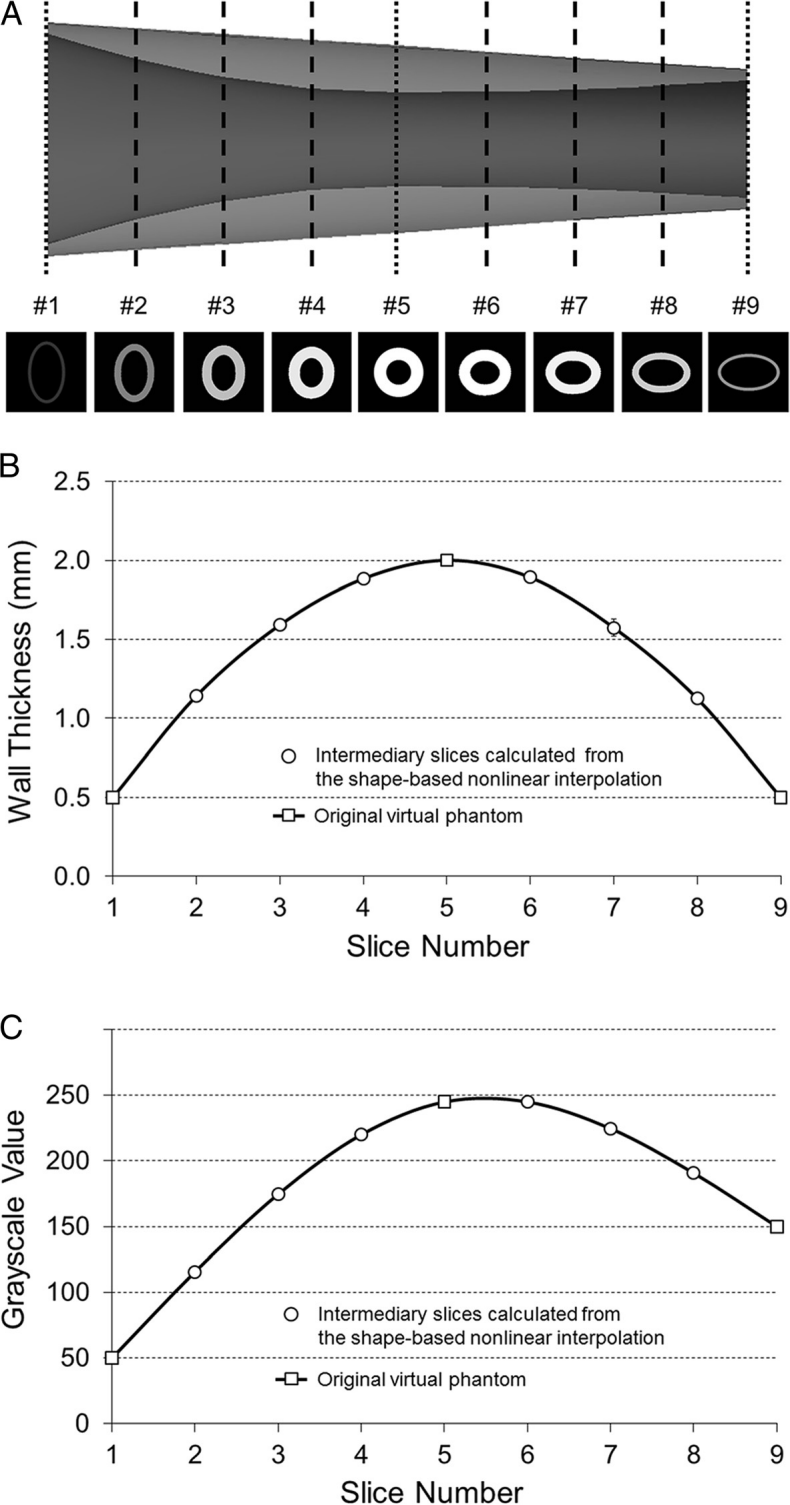
**Validation studies using the virtual vascular phantom. (A)** Original input slices of the phantom and subsequent intermediary slices created using the shape-based nonlinear interpolation method. (**B**) Changes in wall thicknesses. (**C**) Changes in grayscale values.

Changes in wall thickness and grayscale value of the intermediary slices created using the shape-based nonlinear interpolation method were determined and compared to the original cross-sectional slices at the corresponding location of the vascular phantom (Figure 5). Three intermediary slices (#2, #3, #4, #6, #7, #8) were calculated between each pair of the original vascular phantom slices (#1, #5, #9).

Both wall thickness and grayscale values of the intermediary slice images demonstrated a smooth transition over the original vascular phantom slices images (Figure 5A). In Figure 5B and C, the squares indicate the wall thicknesses and grayscale values of the original phantom slices and the circles demonstrate those of the intermediary slices created by the shape-based nonlinear interpolation method. The difference in wall thickness between the calculated intermediary slices and the corresponding original phantom slices was 0.013 ± 0.019 mm. These validation studies indicate that the natural cubic spline interpolation algorithm has been successfully incorporated to create intermediary slices that contain physiologically accurate shapes, wall thicknesses, and grayscale values of a vascular structure with varying morphology and acoustic backscatter characteristics.

### Conventional pixel-based interpolation vs. shape-based nonlinear interpolation algorithm

**Figure 6:**
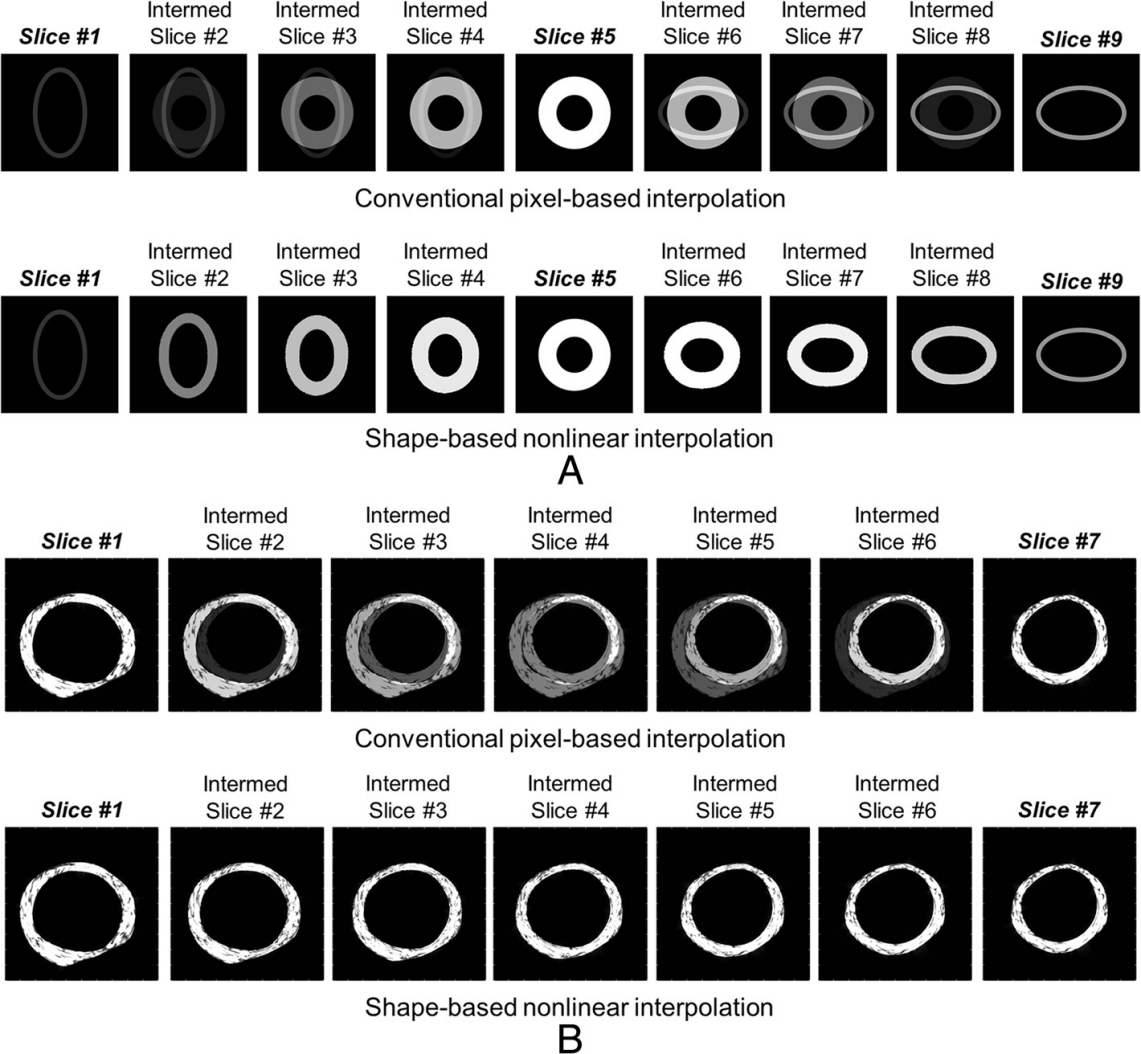
**Validation studies using the femoral artery IVUS data. (A)** Intermediary cross-sectional slice images of the vascular phantom using the conventional pixel-based interpolation (top) and the shape-based nonlinear interpolation methods (bottom). (**B**) Intermediary IVUS slice images of the femoral arterial segment: the conventional pixel-based interpolation method (top) and the shape-based nonlinear interpolation method (bottom).

The original and intermediary cross-sectional slice images of the vascular phantom created by the conventional pixel-based interpolation and our shape-based nonlinear interpolation methods are demonstrated in Figure 6A. The conventional pixel-based interpolation displayed physiologically unrealistic intermediary slice images containing overlapped shapes and grayscale values of the neighboring original phantom slices (top). The shape-based nonlinear interpolation method provided smoothly reconstructed vascular phantom structure borders filled with uniformly distributed grayscale values in the intermediary slice images (bottom).

### Intermediary IVUS image generation of the femoral artery

Figure 6B demonstrates the original 2D IVUS slice images (#1, #7) from the femoral arterial segment and the subsequent intermediary slice images (#2, #3, #4, #5, #6) using the conventional pixel-based interpolation and our shape-based nonlinear interpolation methods. Five intermediary slice images were generated between the adjacent original IVUS images. The conventional pixel-based interpolation method resulted in unrealistic intermediary slice images of the arterial segment (top). The shape-based nonlinear interpolation method provided five intermediary IVUS slice images with physiological arterial segmentation and also provided appropriate acoustic backscatter information (bottom). The shadow effect observed in the intermediary slices generated by the conventional pixel-based interpolation algorithm did not appear in those created by the shape-based nonlinear interpolation method.

### Volumetric 3D IVUS visualization of the femoral artery

**Figure 7:**
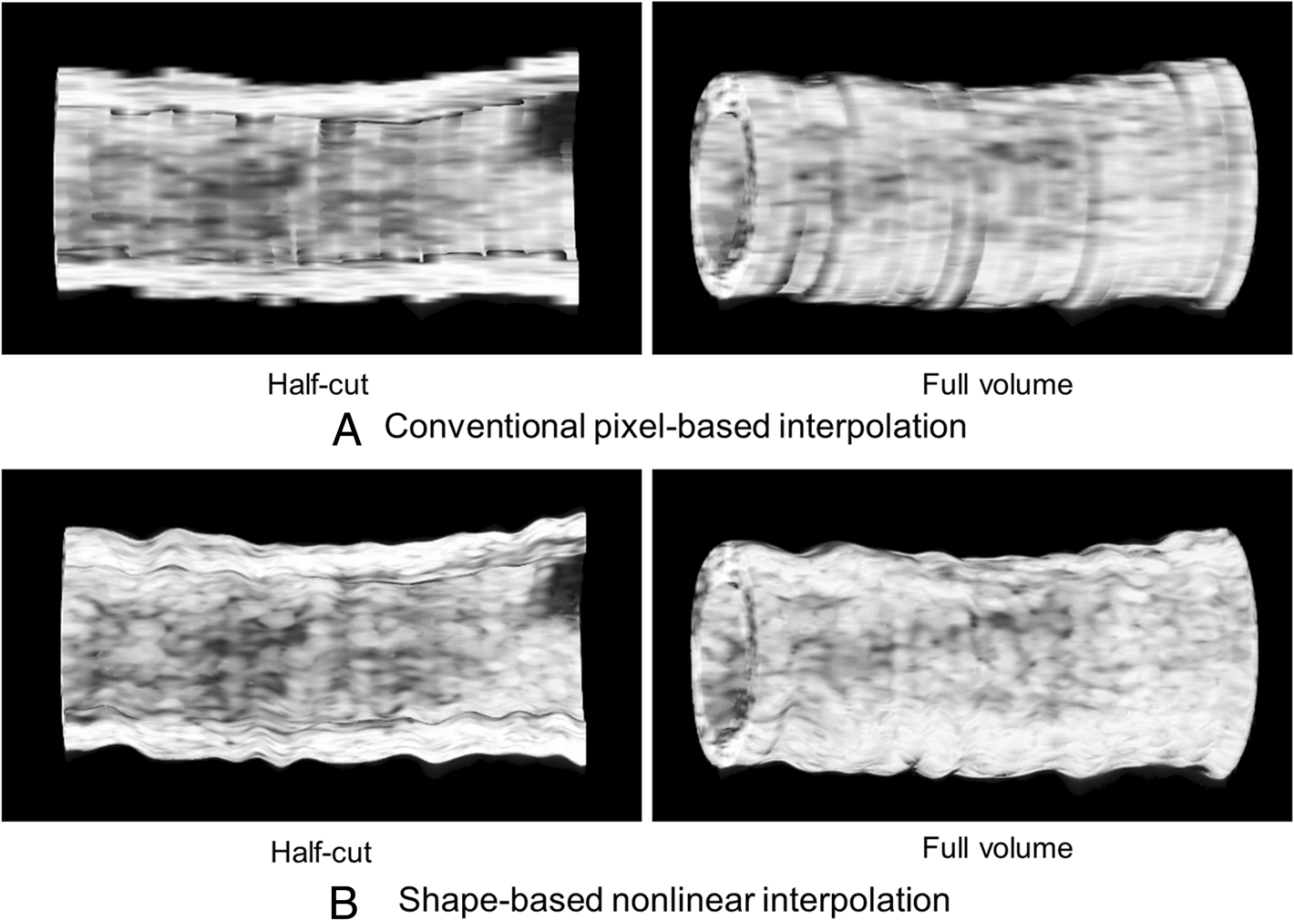
**Volumetric 3D IVUS visualization of the arterial segment.** (**A**) Conventional pixel-based interpolation method. (**B**) Shape-based nonlinear interpolation method.

Volumetric 3D IVUS images of the femoral artery reconstructed using the conventional pixel-based interpolation and the shape-based nonlinear interpolation methods are demonstrated in Figure 7. IVUS signal data with a total of 256 slices over the femoral arterial segment containing both the original IVUS slices and interpolated intermediary IVUS slices were utilized to create the volumetric 3D IVUS arterial models. Our shape-based nonlinear interpolation demonstrated improved volumetric 3D visualization of the arterial structure and more realistic acoustic backscatter distribution compared to the conventional pixel-based interpolation method. While the half-cut 3D IVUS image from the conventional pixel-based interpolation method displayed discontinuous luminal surface boundaries and uneven acoustic backscatter distribution, smoothly reconstructed luminal surface of the arterial segment with physiologically more appropriate acoustic backscatter distribution was observed with the shape-based nonlinear interpolation method.

**Figure 8:**
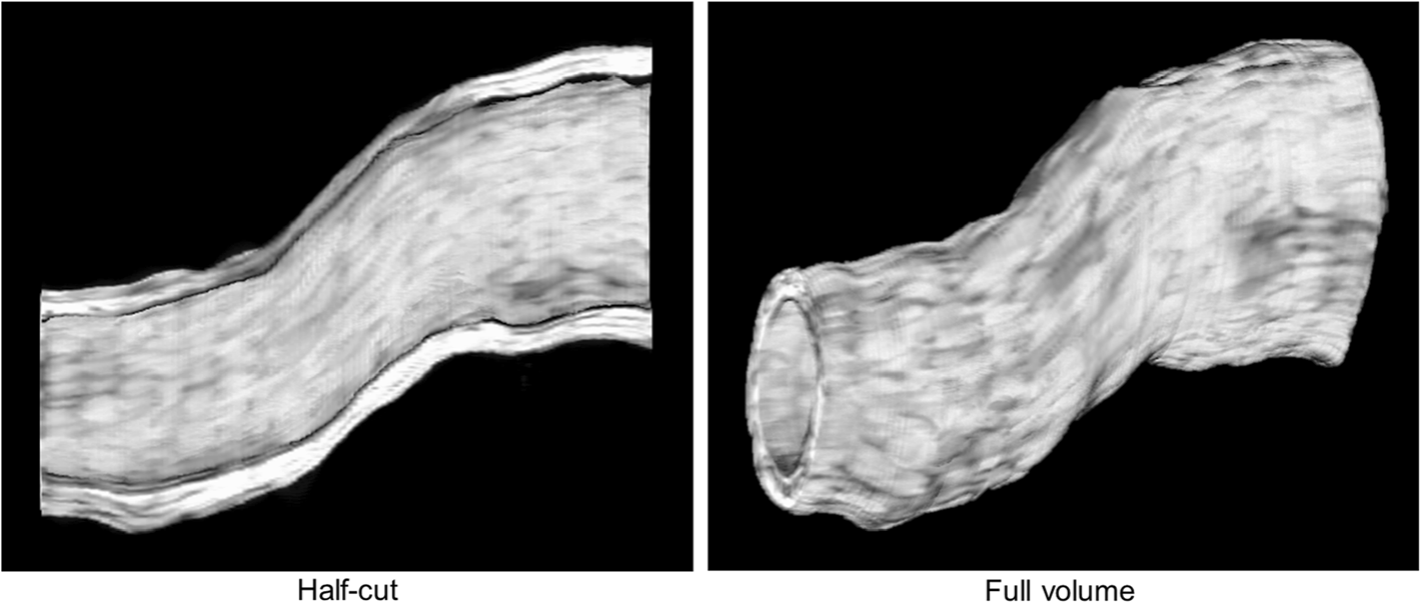
Volumetric 3D IVUS visualization of a curved arterial segment with the shape-based nonlinear interpolation method demonstrating its applicability to 3D IVUS reconstruction for coronary arteries.

Figure 8 demonstrates volumetric 3D visualization of the IVUS data incorporated into a curved pullback trajectory. The IVUS image data were successfully implemented into the curved pullback trajectory and the volumetric 3D IVUS visualization displayed smoothly reconstructed luminal and outer surfaces of the arterial segment with correctly interpolated acoustic backscatter information.

## Discussion

Conventional cross-sectional 2D image data from most commercial IVUS imaging systems provide limited comprehensive information pertaining to complex spatial distribution of arterial morphology and acoustic backscatter information. Volumetric 3D IVUS data can provide a powerful tool to overcome this problem. Accurate 3D visualization of both the morphology of the arterial structure and acoustic backscatter distribution inside the arterial wall will help better understand the extent and stage of atheroma formation [11]. Previous work has developed 3D visualization methods using 2D IVUS images extracted from analog video tapes [3,6-8]. Although there have been great advances in the development of IVUS technologies including phase-array catheters and digital data processing, little emphasis was placed on improvement of 3D IVUS visualization.

In the present study, we presented a novel volumetric 3D IVUS visualization strategy with an intermediary IVUS slice generation method using the shape-based nonlinear interpolation. The non-isotropic voxel dimensionality from 3D IVUS data due to poor spatial resolution can be appropriately resolved by this interpolation method. Moreover, our shape-based nonlinear interpolation algorithm can be incorporated with both 2D IVUS pixelated images and IVUS signal data which provide much higher resolution than pixelated image data alone. Another important feature of our shape-based nonlinear interpolation method is the ability to create physiological acoustic backscatter distribution information in the intermediary slices generated between neighboring 2D IVUS slices. The nonlinear interpolation for acoustic backscatter determination in 3D space will be helpful particularly in ultrasound molecular imaging. In molecular imaging, a variety of targeted ultrasound contrast agents are utilized to detect various molecular component expressions. Improved 3D IVUS demonstration of targeted ultrasound contrast agents in the arterial lesion is expected with this nonlinear interpolation strategy for acoustic backscatter determination over the arterial structure.

For coronary artery imaging, IVUS can be combined with angiography or computed tomography (CT) allowing better determination of arterial curvature in 3D space [12,13]. We made the assumption of no curvature in the validation and femoral artery studies; 1) to better explain the algorithm of our shape-based nonlinear interpolation method, 2) to better demonstrate representative intermediate slices for easy comparison with the neighboring original IVUS images, and 3) to apply the method to *in-vivo* IVUS data of a femoral artery that was relatively straight with little curvature unlike coronary arteries. We created a curved trajectory and incorporated the IVUS data into a curved pullback trajectory to demonstrate the practical applicability of our shape-based nonlinear interpolation method to 3D IVUS reconstruction of curved arteries. The IVUS image data were successfully implemented into the curved pullback trajectory. This indicates that the shape-based nonlinear interpolation method can be fused with biplane angiography or CT, providing improved 3D visualization and acoustic evaluation of coronary arteries in 3D space.

There are some limitations with the present study. A semi-automated image segmentation algorithm was utilized to trace the inner and outer arterial borders in cross-sectional 2D IVUS slices. *In vivo*, it is a well-known inherent problem that there could be axial and rotational offsets of IVUS catheter probe while pullback process during IVUS imaging [14,15]. We assumed that there was no axial and rotational movement of the catheter in the arterial segment. Despite these assumptions, correction algorithms for axial and rotational movements can be utilized prior to our shape-based nonlinear interpolation method. This will result in creating catheter movement-corrected intermediate IVUS slices.

## Conclusions

We have successfully developed a novel volumetric 3D IVUS reconstruction algorithm using a shape-based nonlinear interpolation method to generate intermediary IVUS slices. *In vitro* validation and *in vivo* comparative studies with the conventional pixel-based interpolation method demonstrated more robustness of the shape-based nonlinear interpolation algorithm in determining intermediary 2D IVUS slices and volumetric 3D IVUS visualization. This novel 3D IVUS visualization strategy has the potential to improve ultrasound imaging of vascular structure information, particularly atheroma determination. Improved volumetric 3D visualization with accurate acoustic backscatter information can help with ultrasound molecular imaging of atheroma component distribution.

## Abbreviations

2D: Two-dimensional; 3D: Three-dimensional; IVUS: Intravascular ultrasound; ROI: Regions of interest.

## Competing interests

The authors declare that they have no competing interests.

## Authors’ contributions

YR carried out the algorithm design and implementation, and drafted the manuscript. DDM participated in the design of the study and coordination. HK conceived of the study, designed the study, and contributed to discussions and suggestions to complete the manuscript. All authors read and approved the final manuscript.
